# Performance and self-perceived competencies of enrolled nurse/midwives: a mixed methods study from rural Tanzania

**DOI:** 10.1186/s12913-018-3096-8

**Published:** 2018-04-11

**Authors:** Edith A. M. Tarimo, Gustav Moyo, Happy Masenga, Paul Magesa, Dafroza Mzava

**Affiliations:** 1 School of Nursing, Muhimbili University of Health and Allied Sciences (MUHAS), P O Box 65004, Dar es Salaam, Tanzania; 2 Ministry of Health Community Development, Gender, Eldertly and Children (MoHCDGEC), P O Box 9083, Dar es Salaam, Tanzania; 3 Institute of Allied Health Sciences, MUHAS, P O Box 65004, Dar es Salaam, Tanzania

**Keywords:** Performances, Competencies, Enrolled nurses, Midwives, Tanzania

## Abstract

**Background:**

Tanzania is experiencing a severe shortage of human resources for health, which poses a serious threat to the quality of health care services particularly in rural areas. Task shifting has been considered a way to address this problem. However, since a large percentage of health care providers in rural setting is comprised of Enrolled Nurse/Midwives (ENMs), most of the health care tasks are shifted to them. This article analyzes the performance and self-perceived competencies of ENMs at the dispensary level; the lowest health facility in Tanzania. Performance refers to routine duties performed by ENMs, and self-perceived competence means self-perceived proficiency in performing nursing/midwifery and medical duties.

**Methods:**

This was a mixed methods study conducted in rural Tanzania. A purposeful sample of twelve (12) informants (six ENMs; two Community Leaders [CLs] and four Dispensary In-charges [DIs]) was recruited for semi-structured interviews. The interviews were supplemented with quantitative data from 59 ENMs. Both thematic and descriptive analysis approaches were used.

**Results:**

Three themes emerged: (1) ‘*Approval of the performances of ENMs in meeting community health needs’* underscores important services the community members got from ENMs at dispensaries. (2) ‘*Experienced difficulties of meeting community health needs’* indicate the problems ENMs encountered while providing services to the community. In striving to serve a large number of demanding clients without adequate medical equipment and supplies, sometimes the ENMs ended up with prescription errors (3) ‘*Appreciating the performances and competencies of ENMs’* shows the acknowledgement of community members towards ENMs’ performance and competencies within and beyond their scope of practice. The community members as well as ENMs and their supervisors knew that ENMs must sometimes provide care that is outside their scope of training and competency. Overall, the performance among ENMs above 38 years of age (*P* < 0.05) and participants of professional development courses (*P* < 0.01) was high.

**Conclusions:**

The results highlight performance and self-perceived competencies of ENMs in struggling to meet community health needs. Additionally, these results highlight the health care system shortfalls in supporting and developing an adequate number of qualified health care professionals so that health care needs of all citizens, including those in rural areas, are met.

## Background

Sub-Saharan Africa (SSA) faces multiple challenges, the greatest of which is a shortage of skilled health care workers. Although the region bears over 24% of the global disease burden, it is home to only 3% of the global health work force [1]. In SSA, the scope of the health workforce crisis is alarming in the sense that, there is insufficient adequately trained human capacity of all cadres [1]. In most parts of SSA, the crisis is particularly severe in rural areas where most health workers prefer not to work. Also, it has been noted that the most common factors that pull health workers, including nurses, away from jobs are the lack of incentives and amenities, as well as limited opportunities for career progression [2, 3]. These factors are mostly found in rural parts of low-income countries. Nevertheless, sustainability of health workers in the rural setting is a problem even in high-income countries such as Canada [4], implying that the crisis of health workers is universal.

In Tanzania, the human resources for health per population of 47,524,276 are as follows: one (1) medical doctor per 20,010 populations while WHO recommends one (1) medical doctor per 4000 population, and one (1) nurse /midwife per 1374 populations while WHO recommends one (1) nurse/midwife per 492 populations. Also, one (1) Clinical Officer/Clinical Assistant is currently serving 4809 populations. These ratios are based on 2015 Population Estimates Data by National Bureau of Statistics (NBS) [5]. WHO recommends optimizing available health workforce roles to improve health care services so as to attain the Millennium Development Goals (MDGs) in maternal and child health. As such, it is expected that most of the tasks of other health care workers in rural and remote areas will be shifted to nurses and midwives [6]. In Tanzania, these are often enrolled nurses and midwives (ENMs). ENM in this study refers to a category of licensed nurses and midwives who have received two years formal training at the certificate level. This two year training is a competency based education and training (CBET) system for nursing and midwifery. The curriculum is under National Technical Award (NTA) level 4–6 where the learner should complete all competencies in modules form. Most of the ENMs complete four years of secondary school prior to enrolment into the ENMs’ certificate program.

In Tanzania, out of 31,618 Nurses and Midwives, about 18,255 are enrolled nurses and midwives [7]. After being awarded a two years training certificate, the ENMs are employed by the government, faith based organizations, and private health facilities, often deployed to work in rural dispensaries. Nursing and Midwifery practice is guided by a Manual for Nursing Practice in Tanzania [8], and the Standard of Proficiency for Midwifery Practice in Tanzania [9]. Although the competence descriptor for ENMs require the holder to apply skills and knowledge at a routine level under supervision of registered licensed nurses and midwives [10], in most circumstances this arrangement is not feasible due to the shortage of registered nurses/midwives in the rural settings. As a result, most tasks in rural settings are performed by ENMs who may not have adequate competencies to meet the community needs as they strive to deliver dependable care. Therefore, this article analyzes the performances and self-perceived competencies of ENMs working at dispensary level in rural Tanzania. In this article, performance refers to routine duties done by ENMs at the respective dispensaries, and competence refers to self-perceived proficiency in performing nursing/midwifery and prescriptions.

## Methods

### Design

This was a mixed methods study where both qualitative and quantitative designs were used. The quantitative was preceded by qualitative design.

### Setting

The study was conducted in rural dispensaries in the Coastal Region. Administratively, the region is divided into six (6) districts namely Kibaha, Mafia, Kisarawe, Mkuranga, Rufiji and Bagamoyo. The majority of people in these districts are small scale farmers with very low incomes. Four (4) districts among the six (6) namely Kisarawe, Mkuranga, Rufiji and Bagamoyo were conveniently selected for this study due to proximity and availability of funds to carry out the study. Kisarawe has one (1) hospital, three (3) health centres and twenty five (25) dispensaries. Mkuranga has one (1) hospital, five (5) health centres, and thirty four (34) dispensaries. Rufiji has two (2) hospitals, five (5) health centres and sixty three (63) dispensaries. Bagamoyo has one (1) hospital, five (5) health centres, and sixty six (66) dispensaries.

### Study population

The study population consisted of Enrolled Nurse /Midwives, dispensary in charges who were also the Clinical Officers and Community Leaders who served as local government leaders in the villages where the dispensaries were located.

### Study sample and sampling procedure

#### Qualitative sample

A predetermined sample of twelve (12) informants was recruited: Six Enrolled Nurse Midwives (ENMs); four Dispensaries In charge (DI); and two Community Leaders (CL) were recruited.

#### Sampling strategy

Purposeful sampling was employed to recruit potential informants. The district medical officer and local government leaders facilitated recruitment of the potential study participants into the study. After obtaining permission from the district authorities, the authors introduced themselves to potential informants and asked for their consent to take part in the study. Only ENMs and DIs who had worked for six months or more in the rural dispensary and CLs who had lived in the respective community for one year or more were considered because they were likely to have extensive experience in the study setting. All informants were considered information rich cases.

#### Quantitative sample

Based on availability of ENMs during data collection period, a convenient sample of 59 ENMs was recruited from the dispensaries within the selected districts. The quantitative study was deemed important to supplement the qualitative results.

#### Sampling strategy

One month after completion of the qualitative data collection and analysis, a convenient sample of 59 ENMs was recruited into the quantitative part of the study on an availability basis. The authors obtained permission from the district authorities to gain access to the dispensaries. Only ENMs who were available during data collection and fulfilled the inclusion criteria were asked for consent.

### Data collection technique and tool

#### Qualitative data collection tool

The interview guide was developed based on researchers’ experiences about the community health needs and practices of ENMs in various settings. Also, the questions for the interview guide were determined by the type of interviewee and intended purpose of information to be sought.

Data collection started after obtaining ethical clearance from the Institutional Review Board of Muhimbili University of Health and Allied Sciences (Ref. NO. MU/DRP/AEC/Vol.XVIII/116). The authors described the purpose of the study to all potential participants and obtained their consent to participate in the study. A semi-structured interview guide with face- to- face questions in Kiswahili, the Tanzanian national language, was used to collect data.

For the ENMs, the interview guide consisted of: socio-demographic status; daily responsibilities in the respective dispensaries, common community’s health needs, areas the ENMs thought they did not get exposure to, but the community needed them to have that expert. Also the ENMs were asked about the difficulties they were experiencing during delivery of services to surrounding community including patients.

For the DIs, in addition to their socio-demographic characteristics, they were asked about: who does what among health care providers in their respective dispensary, the common procedures performed by ENMs, their opinion on general performance of ENMs, and their suggestions to improve the competencies of these ENMs.

For the CLs, on top of socio-demographic characteristics, they were asked about the community’s health service needs from the dispensary; the common services the community needed from the dispensary, and what would the community expect from the nurses and midwives at the dispensary level. Two pairs of authors conducted the interviews. Audio recorders were used to record the conversations. While one author was interviewing, another one was managing the audio-recorder and taking notes. All interviews were conducted in privacy based on the informants’ preference. The interviews took between 18 and 28 min. Although we used a pre-determined sample, the collected information became repetitive signalling data saturation.

#### Quantitative data collection tool

The questionnaire was developed using information from the primary health care (PHC) document, Manual for Nursing Practice in Tanzania (basic nursing procedures) and Standards of Proficiency for Midwifery Practice in Tanzania. Additional questions were formed from the qualitative findings.

Content and face validity were determined through the use of two collaborators, and two independent reviewers who reviewed the tool along the whole study proposal. Also two senior ENMs who were not part of the study reviewed the questionnaire as part of pre-testing. All reviewers provided feedback which was used to adjust the questionnaire before use in the field.

Therefore, data was collected by using a pre-tested structured questionnaire to assess performance and self-perceived competence of ENMs. The questionnaire consisted of socio-demographic characteristics, a series of questions constructed from the elements of PHC, Manual for Nursing Practice in Tanzania (basic nursing procedures) and standards of proficiency for midwifery practice in Tanzania. As planned from the start of project writing, the qualitative data generated constructs that were used to develop additional questions for enriching the questionnaire. Each statement had four options. For performance, 1 = Not performed at all, 2 = Sometimes performed, 3 = Often performed and 4 = Performed all the time, and for self-perceived competence, 1 = Very poor, 2 = Poor, 3 = Good and 4 = Very good. The authors administered an in-person questionnaire to ENMs after obtaining their consent.

### Data analysis

#### Qualitative data analysis

Analysis followed thematic analysis approach [11]. The following steps were followed: The first author listened to all audio-recordings immediately after the field work to get a sense of what the informants communicated. She shared notes with other authors to highlight areas which needed further probing. The probes were used to obtain more data through subsequent interviews. Two research assistants transcribed all interviews verbatim. The first and fourth author repeatedly read all transcripts and independently wrote codes on the margin of each transcript. They repeatedly read all transcripts to identify ideas and patterns. The identified ideas and patterns were organized according to similarities and presented briefly to reflect what the informants said. After organizing the whole dataset, important information in relation to overall research question was reported in the form of patterns (themes) and three themes emerged. The coding process and the preliminary results were discussed in the debriefing meeting between all authors and stakeholders. During the debriefing meeting, one of the stakeholders; a qualitative expert commented in the analysis process. The comments were discussed, agreed upon, and included in the final analysis. All authors reviewed and agreed on the emerged themes.

### Quantitative data analysis

#### Data entry and management

Data from questionnaires was cross-checked to facilitate coding and tabulation of complete datasets. No missing values were recorded. The coding sheet comprised a list of all variables. Shortened variable names were used in the Statistical Package for Social Science (SPSS) version 20 and the responses were coded.

### Data analysis was carried out in three stages

#### Stage one

The frequency distribution of all variables was tabulated. Twenty-one (21) statements assessed both performance and self-perceived competence of ENMs. All statements about provision of nursing / midwifery services in the community and hospital/dispensary, elements of Primary Health Care (PHC), professional development and collaboration, and medical/clinical profession duties were used to assess performance and self-perceived competence amongst ENMs. Each desired response scored 1 and each undesired response scored 0, the median score being 18. The performance was assessed by ‘Not performed or sometimes performed=low performance’ and ‘Often performed or performed all the time=high performance’. The self-perceived competence was assessed by ‘Very poor or poor=low self-perceived competence’ and ‘Good or very good=high self-perceived competence’. Scores for both performance and self-perceived competence were therefore dichotomised as ‘High performance/High self-perceived competence’ for those who scored above the median (median score > 18) or ‘Low performance/Low self-perceived competence’ (median score below or on a par with median (median score ≤ 18).

#### Stage two

The authors analysed performance and self perceived competences of ENMs and examined the association between these variables and socio-demographic characteristics, work experience, participation in continue development courses, and use of available supportive supervision. Cross tabulation was performed between selected variables.

#### Stage three

Chi square test was performed to detect the relationships between variables. A *P*-value of < 0.05 was regarded as significant.

## Results

### Qualitative results

#### Socio-demographic characteristics

Twelve (12) informants participated in the study. The ages ranged between 24 and 53 years (mean age 39, SD 8.8). The majority had attained four years of secondary education. In a Tanzania system of education, this is secondary education prior to high school. All Community Leaders (CLs) had lived in the same community for more than five years. All Enrolled Nurses/Midwives (ENMs) had four years of secondary education and had worked for more than two years in the respective dispensaries. The DIs had worked for more than six months in the respective dispensaries and all had attained secondary education (Table 1).

**Table 1:** Socio-demographic characteristics of informants. Qualitative Part

SN	Participants’ category	Age	Sex	Education Level	Experience in the respective community
1	Enrolled Nurse (01)	43	F	Form IV	5 years
2	Enrolled Nurse (02)	32	F	Form IV	8 years
3	Enrolled Nurse (03)	24	F	Form IV	2 years
4	Enrolled Nurse (04)	33	F	Form IV	5 years
5	Enrolled Nurse (05)	35	F	Form IV	5 years
6	Enrolled Nurse (06)	40	F	Form IV	11 years
7	CL (07)	45	M	STD VII	> 10 years
8	CL (08)	44	F	Form IV	10 years
9	DI (09)	53	M	Form IV	9 years
10	DI (10)	32	F	Form IV	5 years
11	DI (11)	38	F	Form VI & University	6 years
12	DI (12)	53	F	Form IV	4 years

#### Themes

Three (3) themes emerged: The first theme, ‘*Approval of the performances of ENMs in meeting community health needs’* underscores important services the community members got from ENMs at the dispensaries. The second theme, ‘*Experienced difficulties of meeting the community health needs’* indicates the problems encountered while providing services to the community. The third theme, ‘*Appreciating the performances and competencies of ENMs’* shows the acknowledgement of community members towards ENMs’ performance and competencies within and beyond their scope of practice.

### Approval of the performances of ENMs in meeting community health needs

Through experiences of living in the same community, the CLs described their observations regarding performances of ENMs. They said that majority of pregnant women and children from within and outside the village were getting antenatal, obstetric delivery, family planning, vaccinations and medical services from nurses [ENMs] at the dispensaries. They recognized that ENMs were referring some clients/patients to a higher level health facility for further management or where they could get services which were not available at the respective dispensary.

Although most ENMs were residing far away from the work place, the CLs noted that these nurses were consistently responding to the health needs of people in the community. They said:

“When a woman in labour pains is brought at night, the nurses will be called from their home. We have two guards here; they call the nurses when they see clients around…if they live far, the guards will call the motorcycle owner to pick up the nurse; the nurse will immediately arrive to serve the woman…when clients are on nocturnal injections, the nurses attend them… Nurses are good. Whenever they are called they come immediately” (CL 08).

Also the CLs commended nurses for good work, commitment to save lives, warmth, and friendliness towards clients. They said:

“They [nurses] perform all duties… Although this is a dispensary where services should end on Friday, but even on Sundays, the nurses provide services … on Monday there are many clients, and all of them get services. Nobody will tell you that he/she missed services because of coming after working hours…”(CL 07).

The DIs perceived ENMs as crucial group in meeting the community health needs. They said that the community enjoys the ENMs’ services because they recognize them as doctors [clinical officers]. Also some DIs added that a number of experienced ENMs are experts in the sense that they teach the clinical officers what to do:

“From her [ENM] knowledge, there are other aspects that I can learn from her [ENM]… She knows things which I don’t know. For example in the labour ward, I am not doubtful at all because I know she can handle everything. When she says, ‘Doctor, this woman will deliver without complications, truly, the woman will deliver without problems’. If she suggests sending the patient further [referral], you [I] do that. Even on treatment issues, she advises me the type of medicines to give…” (DI, 11).

Another DI complemented:

“She [EN] takes care of the cases that are within her scope. The diseases that even a layman can understand and what type of medicines ones should take. For example malaria, cough, she knows according to her work experience…she knows to treat urinary tract infections [UTIs]…she can order malaria test, Hb [amount of blood] plus urine tests” (DI 12).

Overall, the DIs valued the ENMs services to the community and stated that ENMs have confidence in performing their duties in the sense that even the community appreciates the services they get as detailed bellow:

“They [community] know what they [ENMs] do. For example, I had never seen a patient returning, presenting with complaints that I was attended by so and so and things went wrong… Overall they [ENMs] are knowledgeable. They know when the patient presents with this, what to give …When they attend patients, the patients get cured...” (DI, 11).

Another DI added:

“I had never received complaints about her [ENM]. .. The way I see it, when I travel she is responsible for all duties [Nursing/Midwifery and Medical]. She treats patients and the RCH [reproductive and child health] services continue to run smoothly” (DI 12).

The ENMs affirmed that they often provide reproductive and child health services, antenatal care, prevention of mother to child transmission, and vaccinations for children., They also said they provide counselling services on family planning, STIs and HIV testing, and obstetric deliveries. As a way of striving to meet the community health needs, the ENMs said they arranged a working schedule to allow consistent service provision to clients who arrive at the clinic after working hours:

“Everybody has a weekly schedule to assist deliveries at night after working hours. We often start at 7:30AM and work until 3:30PM, but then continue to work throughout the night. Tonight, they [guards] call us, the guards call us and tell us ‘there is a woman here who is expecting to deliver’… Per day you may assist 5-6 deliveries” (ENM 03).

### Experienced difficulties of meeting the community health needs

Most ENMs stated that they extended working hours because of striving to meet the needs of the large population that they had to serve. They stated that they provided maternal and child health services to a large number of clients. However, they were overwhelmed because in most cases they were few and the number of clients to be served was so large. One ENM said:

“There are those who come to start clinic [ANC], you have to carry out Prevention of Mother To Child Transmission [PMTCT] investigations. If there is one who is positive [HIV], there is the issue of registering her on option B plus regime and opening a file; things like that. Per day you may end up with six pregnant women; that is too much. Then there are follow up cases…The population is large…our plan is to serve 13 women per month, but currently we are enrolling more than 38!” (ENM 01).

Another ENM commented on the duties and prolonged working hours:

“I have too much to do. For example, as I am working at this dispensary, it will reach time when I am to go home ..., just before taking off my uniform, I was called back to attend a woman in labour pains…After assisting her, another woman came in. It is not surprising to spend the whole night, and in the morning I have to start my regular shift” (ENM 06).

In addition they added:

“Responsibilities are more than what I expected because sometimes I may plan to do this perfectly, but I encounter challenges. Therefore, I find myself trapped in responsibilities that extend beyond my capacity” (ENM 04).

While striving to meet community health needs, the ENMs faced challenges such as negative comments from clients. One ENM said:

“At night you may receive a woman who is in labour pains, but you have nothing [delivery equipment]. A small percent of women understand the situation, but others feel that we are being rude to them. They [clients] think that we receive medical equipment plus medicines and sell them! If you refer to the registry, no Oxytocin [special medicine for inducing labour] has been supplied here [at the dispensary]!” (ENM 03).

Also some of the ENMs experienced situations where clients were not satisfied with available services at the dispensaries. They said some of them spent a long time at the dispensary only to find out that the medicines they were prescribed were not available. On top of that, they said that the environment was not very conducive to provide services, yet they managed to provide most of the services to the clients:

“The environment in this dispensary does not offer privacy and safety. Somehow it is a problem. Women want to give birth at this dispensary, but the space is very small…the delivery room is small and there is no electricity… No delivery kit; medicines do not arrive on time… Therefore, clients are not comfortable…We may lose [death] clients” (ENM 01).

Most of the ENMs were distressed with the irregular supervision from the regional or district office, inadequate equipment, and essential medicine to support service delivery. Some women had no money even to buy gloves. The ENM added.

“It is painful because some clients do not have money. The majority of women are not financially capable. When you look at their status you can realize that; that is the situation; we don’t have a mechanism to assist them…” (ENM 03).

Sometimes the ENMs complained that they did not have the necessary knowledge on how to handle various lab investigations and prescriptions, yet clients wanted to get all services from them.

One DI shared her experience in this:

“Where I practiced before, it [prescription errors] happened several times. They [ENMs] were giving medicines without investigation results. The medicines were the ordinary ones. But you may find a client [community member] complaining that I told her [ENM] this but she gave me that. This is due to their [ENMs’] level of understanding…” (DI 12).

Another DI was concerned about the competences of ENMs. She said:

“She might come across complicated cases and she needs to make decision by herself… my worries about her decision is that she might end up with inappropriate decision!” (DI 11).

Despite the fact that ENMs were striving to meet community health needs with constrained resources, the CLs said there were complaints about nurses from community members. The CLs stated that since they are involved in management and sustainability of dispensaries, they understand how much nurses strive to meet the clients’ needs with limited resources:

“The main problem is availability of medicine; medicines are not adequate… obstetrical delivery services are carried out here; and there is no water supply. Relatives are forced to bring buckets of water when they escort a woman who is in labour pains… It is a challenge especially when there is shortage of water supply in the hospital…The lab has no equipment to carry out all investigations; malaria test yes, but not blood levels [Haemoglobin level]” (CL 08).

In most circumstances, the CLs observed that ENMs were serving a larger population than anticipated and were overwhelmed with the challenges they encountered. The DIs felt the need to have more ENMs to serve the targeted community. In a similar vein, the ENMs noted that the community needs were far beyond their scope of practice and available resources.

### Appreciating the performances and competencies of enrolled nurses and midwives

Task shifting emerged as a difficult process where all ENMs were trapped in between performing their core duties as ENMs and medical/clinical duties, which were beyond their scope of practice. Despite the fact that most ENMs were already overstretched by their own roles, they said they were forced to shoulder medical professional duties in order to meet the health needs of the community. For example one ENM said:

“Sometimes when the doctor [clinical officer] is not available, perhaps if he/she is attending a meeting/seminar, I take over his/her responsibilities…” (ENM 01).

One DI complemented:

“At the dispensary, a nurse [ENM] performs all duties. I mean in addition to her/his duties such as attending pregnant women and children, she/he treats patients if I am not around… She/he will treat patients and perform all her/his duties as usual…” (DI 11).

Although most of the ENMs said they practiced within and outside their scope, some of them referred serious cases:

“I practice according to my knowledge. I attend those clients whom I can manage within my scope. For example, if the client presents with fever, I take the body temperature, I take blood samples for malaria test; if it happens that he/she has malaria, I give him/her malaria dose. If it happens that he/she presents problems beyond my knowledge, I refer him/her to a large hospital” (ENM 03).

The majority of ENMs said that they had worked for more than two years. However, they admitted inadequate knowledge in serving pregnant women who were infected with HIV. Also, they said during their training they were not exposed to treating this type of clients. They desired to get exposure in the medical training.

Overall, most ENMs were trapped in task shifting without a willingness to do so.

Some ENMs recommended having additional training in order to shoulder medical duties in a professional manner. They said since they were doing medical duties, they needed training to avoid harming the patients. Nevertheless, the ENMs expressed appreciation of their own efforts in gaining experience through team work. They stated that discrepancies in treating patients were common, but they often corrected the mistakes as they arise. One ENM said:

“When the patient complains of this, you give that; we believe there might be side effects that we may not be aware of, but the doctor would know…” (ENM 04).

Another ENM complemented:

“You know I have gained experience while working here…the more I work, the more knowledge I gain. Therefore I don’t see any new situations” (ENM 01).

The DIs stated that they usually mentor those ENMs who come directly from training so that they can perform their duties and shoulder medical duties. However, they cautioned that patients might have been treated inappropriately, but because of their ignorance about types of medicines they are supposed to take, they never reported [about inappropriate treatment]. Overall, the DIs perceived that community members were not aware of the difference between medical officers and nurses [ENMs]; what they care about the most is to get cured from the diseases and illnesses from which they suffer.

Despite mentoring efforts among ENMs, the CLs said they came across discrepancies that made them realize that there might be a different between a nurse and a doctor. This happened when a clinical officer was not available and then the ENM had to prescribe medicines to patients. The CL narrated his personal experience with ENMs’ performance:

“When you bring a sick child at the health centre [dispensary], the child may be given wrong medicine…that is an error … It happened to my child. The child had to be admitted in a referral hospital. Later on the doctor who attended the child ordered the child to be given milk, stating that the child was overdosed…” (CL 07).

The DIs stated that they understood that professional expertise differs between them and ENMs; yet due to shortage of human resource, they were forced to ask the ENMs to shoulder medical professional duties.

Additionally the ENMs complained that they faced challenges in shouldering medical duties especially when it came to an expertise that was not covered during their training. In most circumstances, the ENMs did not embrace task shifting. Rather they took it as an obligation that one was supposed to shoulder in order to meet the community’s health needs. However, sometimes they sensed a loss of confidence when performing clinical duties. They said:

“I had never been trained as a medical prescriber…truly I have realized that is beyond my competence… Therefore, practicing as a clinician lowers my confidence, but you [I] do it because it is your [my] obligation to provide service to the clients... What else can you [I] do?” (ENM 01).

Another ENM added how difficult it was for her to shoulder clinical responsibilities. She said she sometimes performed duties which she had never been trained for:

“The duties that I perform are far beyond my competencies which include: making diagnosis and writing prescriptions. In the college, I was not trained to do this …” (ENM 06).

Moreover, the ENMs noted that task shifting was practiced beyond medical territory where health attendants [non-medically trained personnel] were providing health services to patients. They said:

“Here we have medical attendants. We help each other. You know the situation; one person cannot do everything. We have to share the work: one can take care of children [paediatrics], another one provides vaccinations, another one may assess pregnant women and another one can distribute medicines. Things like that …” (ENM 01).

The ENMs understood that health attendants (HAs) were not allowed to provide care to the patients, but they asked them to take part in service delivery under the umbrella of ‘task sharing’ and shortage of trained and qualified nurses. However, they were cautious about the implications if something would go wrong:

“Personally I gained a lot of benefits from them [HAs]. For example, today I am alone. Therefore, they are helping me to assess pregnant women and they are helping to vaccinate…Truly these health attendants assist, but if problems occur, the blame will fall on us who are responsible” (ENM 06).

Other ENMs added that some challenges rose when clinical officers were not available and as nurses they were not able to intervene. For instance, during home visiting, the ENMs said they came across serious cases in the community which would require experts from the medical field. Although they said that they were aware of treatment guidelines such as those for STIs, they were not comfortable to go back and forth to consult treatment guidelines in order to give prescriptions to patients at home. They confessed that they had to carry out medical duties not because they liked to do so, but because of the shortage of staff. They said:

“You may wish to have a qualified person to fix the situation [treat the patient], such things would be perfect. But when you see one practicing beyond his/her scope, it is a burden. Extra efforts are needed because there is either mismanagement or errors. We try to make corrections among ourselves; that is how we move along…what else can we do?” (ENM 01).

Nevertheless, they did what they could to meet the clients’ needs:

“We discuss with the patient if he/she can talk. If he/she cannot we collect information from relatives. We carry out assessments and come up with diagnosis and give medicines” (ENM 04).

Overall the ENMs stated that the skills and knowledge they acquired on provision of home based care during training were not adequate.

### Quantitative

#### Socio-demographic characteristics

A total of 59 participants from four districts in Coastal Region were recruited in the study. Of the 59, 17 (28.81%) were from Bagamoyo; 16 (27.12%) from Kisarawe; 16 (27.12%) from Mkuranga and 10 (16.95%) from Rufiji. The majority of the participants were female (96.6%), between 36 and 45 years old (mean 38, SD 9.8), educated up to four years of secondary education (71.2%) and married (56.6%). Several participants (35.6%) had worked between 16 and 30 months in the current dispensary; (28.8%) had worked as EN/EM between 2 and 5 years and most of them were Enrolled Midwives (64.4%). Of 59 participants, 42 (71.2%) were Christians; the rest were Muslims (Table 2).

**Table 2:** Socio-demographic characteristics. Quantitative Part

Variable	Frequency	Percent
Sex		
Male	2	3.4
Female	57	96.6
Age		
≤ 25	7	11.9
26–35	18	30.5
36–45	19	32.2
46 +	15	25.4
Education		
Primary Education	16	27.1
Ordinary Secondary Education	42	71.2
Advanced Secondary Education	1	1.7
Marital Status		
Unmarried	25	42.4
Married	34	57.6
Work experience (months)		
≤ 12	5	8.5
13–60	21	35.6
61–120	14	23.7
121–240	10	16.9
241 +	9	15.3
Work experience in the current Dispensary (months)		
≤ 12	15	25.4
13–60	22	37.3
61–120	13	22.0
121–240	6	10.2
241 +	3	5.1
Work experience as EN/NM (years)		
≤ 1	5	8.5
2–5	17	28.8
6–10	16	27.1
11–20	11	18.6
21 +	10	16.9
Religion		
Christian	42	71.2
Moslem	17	28.8
Others	0	0

#### The performance and self-perceived competence of ENMs

The performance and self-perceived competence of ENMs were evaluated against ten elements of primary health care namely: 1) Educate patients and community about their health problems; 2) Identifying & controlling prevailing health problems; 3) Food supply and proper nutrition; 4) Provision of safe water and basic sanitation; 5) Provision of family planning services, Maternal & child health care; 6) Provision of Immunization; 7) Prevention and control of endemic disease; 8) Appropriate treatment of common diseases and injuries; 9) Provision of essential drugs; and 10) Provision of mental health care. In addition, the following roles of ENMs per their scheme of service/responsibilities were assessed: 1) Collection of statistical data and prepare report; 2) Provision of home based care and 3) Provide counselling services. Under professional development and collaboration, we assessed 4) Participation in continue professional development courses and 5) Use of available supportive supervision. Furthermore, we assessed the practice of medical professional duties among ENMs as follows: 1) Treating patients when the doctor is not available; 2) Making clinical Diagnosis of patients according to signs and symptoms and write prescription; 3) Requesting for laboratory tests in absence of the doctor; 4) Referring patients to another Health facility according to their conditions; 5) Treating children according to their conditions; and 6) Making a diagnosis and treating patients during home based care. The above components make a total of 21 items which were subjected to analysis as shown below.

#### Levels of performance and self-perceived competence of ENMs

The majority (70% and above) of ENMs demonstrated high levels of performance in relation to: provision of services under seven of ten elements of Primary Health Care (PHC); participation in professional development and collaboration; and providing medical/clinical services. However, low levels of performance (below 70%) were recorded in provision of three elements of PHC. These were food supply and proper nutrition, provision of mental health care, and provision of home based care which falls under their responsibilities. Self-perceived competence was high in relation to all PHC elements and performing medical professional duties (Table 3).

**Table 3:** Selected variables on levels of performance and corresponding self-perceived competencies of ENMs

Variable	Performance	Self-perceived competence
	High (%)	High (%)
Participate in implementation of PHC elements		
Educate patients and community about their health problems	55 (93.2)	54 (98.2)
Identify and controlling prevailing health problems	55 (93.2)	52 (94.5)
Food supply and nutrition	20 (33.9)	17 (85.0)
Provision of safe water and basic sanitation	46 (78.0)	43 (93.5)
Provision of family planning services, maternal and child health care	58 (98.3)	54 (93.1)
Provision of immunization	54 (91.5)	54 (100)
Prevention and control of endemic diseases	53 (89.8)	47 (88.7)
Appropriate treatment of common diseases and injuries	58 (98.3)	54 (93.1)
Provision of essential drugs	58 (98.3)	55 (94.8)
Provision of mental health care	17 (28.8)	12 (70.6)
Additional duties as EN/EMs		
Collect statistical data and prepare report	57 (96.6)	57 (100)
Provide home based care	40 (67.8)	37 (92.5)
Provide counselling services	56 (94.9)	55 (98.2)
Professional development and collaboration		
Participation in continue professional development courses	52 (88.1)	47 (90.4)
Use of available supportive supervision	54 (91.5)	47 (87.0)
Perform Medical / Clinical professional duties		
Treating patients when the doctor is not available	57 (96.6)	55 (96.5)
Making clinical diagnosis of patients according to signs and symptoms and write prescription	57 (96.6)	55 (96.5)
Requesting for laboratory tests in absence of the doctor	53 (89..8)	52 (98.1)
Referring patients to another Health facility according to their conditions	58 (98.3)	55 (94.8)
Treating children according to their conditions	58 (98.3)	55 (94.8)
Making diagnosis and treating patients during home based care	45 (76.3)	38 (84.4)

#### Factors associated with overall performance of ENMs

The association was examined between overall performance of 21 items including socio-demographic characteristics, work experience, participation in professional development courses, and use of supportive supervision. Overall there was a statistically significant difference between levels of performance and age group, level of education, and participation in professional development courses (Table 4).

**Table 4:** Factors associated with overall performance of ENMs

Variable	Overall Performance		*P*-value
Sex	High (%)	Low (%)	
Male	1 (50)	1 (50)	0.746
Female	28 (49.1)	29 (50.9)	
Age			
≤ 38	10 (35.7)	18 (64.3)	0.044
38 +	19 (61.3)	12 (38.7)	
Education			
Primary Education	12 (75)	4 (25)	0.016
Ordinary Secondary Education	17 (39.5)	26 (60.5)	
Marital Status			
Unmarried	11 (44)	14 (56)	0.339
Married	18 (52.9)	16 (47.1)	
Total work experience (months)			
≤ 84	29 (49.2)	30 (50.8)	NA
84 +	29 (49.2)	30 (50.8)	
Work experience in the current Dispensary (months)			
≤ 48	13 (40.6)	19 (59.4)	0.122
48 +	16 (59.3)	11(40.7)	
Participation in continue professional development courses			
Yes	29 (55.8)	23 (44.2)	0.006
No	0 (0.0)	7 (100)	
Use of available supportive supervision			
Yes	28 (51.9)	26 (48.1)	0.187
No	1 (20)	4 (80)	

#### Factors associated with overall self-perceived competence of ENMs

The association between overall competences in 21 items was examined plus socio-demographic characteristics, work experience, participation in professional development courses, and use of supportive supervision. Overall, there was no statistical difference in relation to all aspects in self-perceived competence (Table 5).

**Table 5:** Factors associated with overall self-perceived competencies of ENMs

Variable	Overall self-perceived competence		*P*-value
Sex	High (%)	Low (%)	
Male	0 (0.0)	2 (100)	0.503
Female	17 (29.8)	40 (70.2)	
Age			
≤ 38	6 (21.4)	22 (78.6)	0.184
38 +	11 (35.5)	20 (64.5)	
Education			
Primary Education	6 (37.5)	10 (62.5)	0.278
Ordinary Secondary Education	11 (25.6)	32 (74.4)	
Marital Status			
Unmarried	6 (24.6)	19 (76)	0.344
Married	11 (32.4)	23 (67.6)	
Total work experience (months)			
≤ 84	17 (28.8)	42 (71.2)	NA
84 +	17 (28.8)	42 (71.2)	
Work experience in the current Dispensary (months)			
≤ 48	6 (18.8)	26 (81.2)	0.058
48 +	11 (40.7)	16 (59.3)	
Participation in continue professional development courses			
Yes	16 (32.7)	31 (66)	0.075
No	1 (8.3)	11 (91.7)	
Use of available supportive supervision			
Yes	16 (29.6)	38 (70.4)	0.550
No	1 (20)	4 (80)	

### Comparison of qualitative and quantitative results

The qualitative results correspond to the quantitative results in the following aspects: The qualitative descriptions made by Dispensary In charges (DIs) and ENMs themselves support high levels of performance in medical/clinical professional duties such as giving prescriptions amongst ENMs who took part in quantitative study. The DIs were aware that in their absence, the ENMs were performing medical duties including prescriptions. Similarly, the ENMs confirmed how and why they had to perform medical duties. On the other hand, ENMs demanded additional training to advance their knowledge and skills in medical duties. This wish was highly supported by high levels of performance amongst those ENMS who participated in continue professional development courses.

## Discussion

The results highlight the performance and self-perceived competencies of ENMs at the dispensaries, the lowest level of health care facility in Tanzania. Overall these results show that ENMs actively performed Nursing/Midwifery and Medical professional duties such as prescription orders to meet community health needs. Although most ENMs performed all Nursing/Midwifery and medical/clinical duties, they complained of inadequate knowledge and skills to shoulder clinical duties. Through their experiences in the field, the Community Leaders (CLs) and Dispensary In charges (DIs) noted and reported such knowledge and skills deficiencies amongst ENMs.

The fact that the majority of ENMs were overwhelmed while striving to meet the community health needs implies shortage of staff at the respective dispensaries. In most cases, ENMs appear to provide both Nursing/Midwifery and Medical duties of which the latter is not within their scope of practice. The situation is partly supported by WHO [12] that recommend optimization of available workforce roles in improving health care services. Although task shifting was considered a way to address this issue, additional training is fundamental prior to implementing task shifting in this context. In this study the most alarming challenge is the suboptimal performance of Medical duties the ENMs were subjected to without formal training. The National Technical Awards (NTA) level 4–6 curriculum [10] clearly stipulates the competence descriptor for certificate Nurses and Midwives, that they should be able to apply skills and knowledge at a routine level under supervision of registered nurses as learners. This is contrary to what was found in the field where most ENMs were performing both Nursing/Midwifery and Medical duties in the absence of supervision.

Therefore, it is not surprising that due to lack of staff, available staff were assuming multiple roles and filling gaps which undermined the performance and quality care [13]. It has also been noted that in many Low and Middle income countries, the sustainability and quality of nursing services are threatened by global shortages of healthcare professionals [14].

Since the establishment of training courses for lower cadres in Tanzania, ENMs have been quite instrumental in the Tanzanian health care delivery system. They now occupy the largest proportion of care providers from the district hospitals all the way down to the dispensary level [6]. Although the crisis of skilled health workers in rural areas is not unique for Tanzania, it is necessary to review the performances and competencies of ENMs in order to do justice to the clients. In Malawi, enrolled nurses have similarly been reported to play enormous role in the health systems, particularly in remote areas where it is difficult to get better-qualified health professionals to practice [1]. In order to achieve sustainable millennium development goals, the crisis of human resources for health in rural areas may call upon various top down strategies to rectify the situation. ENMs need to be well trained within their scope of practice in order to provide quality services to the community. In this study, the commonly reported community health needs were related to maternal and child health care. Tanzanian national regulations allow enrolled nurses to assist deliveries under supervision. However, the performance of ENMs in rural settings seems to be hampered by a lack of supervision. Similarly, in Northern Tanzania, the provision of care was hampered by poor supervision from the District level [15]. This situation may endanger the clients if ENMs do not have adequate supervision to cover for the intended health care services.

The fact that several ENMs were concerned about their competencies to meet the needs of the community is worth noting. Since the Tanzanian government vision is to have a healthy community that will contribute effectively to individual development and the country as a whole, the government should ensure ENMs are competent to meet standard community health needs. Continuous professional development may be emphasised to update ENMs in basic clinical skills, and this may increase their job satisfaction. Lack of professional development opportunities among primary health care nurses has been cited as a contributing factor towards job dissatisfaction with regards to their work life [16]. The resulting suboptimal care may be compounded by the lack of skills-based training which confronts those in lower cadres [17]. Nevertheless, the lower cadres, such as auxiliary nurses, have been observed to perform some activities such as counselling during antenatal care [18]. Perhaps, ENMs in the present study would be able to provide optimal care to clients if they were better equipped with the necessary knowledge and skills to do so.

### Limitations and mitigations

The use of qualitative method and a small sample in the quantitative method cannot yield generalizable results. Therefore, the generated results are only applicable to the studied participants. However, we believe these results generate the opportunity to carry out a larger study in the future using a random large sample to enable generalization of results. Also there was a risk of potential bias such as socially desirable responses from interviewees. The authors tried to mitigate this bias by emphasising the purpose of the study and the need for honesty.

## Conclusions

The study findings underscore the actual performance and self-perceived competencies of ENMs while striving to meet the community health needs in the rural part of a low-income country. Despite the difficulties involved in meeting the community health needs, the ENMs serve as an important model in providing health care services under constrained human resources for health. However, what is alarming in this context is the current situation where ENMs are obliged to perform medical duties without formal training. Consequently, the richness of these results could be utilized to review the existing nursing training curriculum, supervision guidelines and recommend short-term strategies which will assist the ENMs to provide optimal services and meet the basic health needs of the community.

### Implications to practice

#### Education

Comprehensive training in provision of mental health care should be incorporated and enriched in ENMs’ curriculum because this is a crucial element of PHC and thus within the ENMs core responsibilities. For in service ENMs, short courses may be introduced as additional training or continued education to master specific aspects.

#### Practice

Supportive supervision guidelines for nurses/midwives at the district level should be revised to ensure consistent monitoring of performance and competence of ENMs at dispensary levels. Also access to more highly trained providers via cell phones could be a short term strategy.

#### Research

Further research using a large random sample is needed to allow for generalization of the results in future. Also based on the current results, intervention studies might provide more insights into their effectiveness in solving some of the problems.

#### Policy

The government through the Ministry of Health, Community Development, Gender, Elderly and Children (MHCDGEC) formally Ministry of Health and Social Welfare (MoHSW) should review the human resource for health and make sure that all Tanzanians get optimal health care services by deploying adequate qualified human resources. Also a clear statement should be incorporated in the existing ENMs’ scheme of service to ensure that all ENMs practice within their scope of practice and when necessary, utilize available referral systems. In addition, the responsible ministry should work towards ensuring acceptable provider/patient ratio.

## Abbreviations

ANC: Antenatal Care
CL: Community Leader
CO: Clinical Officer
DI: Dispensary In charge
ENMs: Enrolled Nurses/Midwives
Form IV: Four years of secondary education prior to high school in Tanzania Education System.
Form VI: High school after four years of secondary school in Tanzania Education System
KOICA: Korea International Cooperation Agency
MHCDGEC: Ministry of Health, Community Development, Gender, Elderly and Children
MoHSW: Ministry of Health and Social Welfare
NBS: National Bureau of Statistics
NTA: National Technical Award
PHC: Primary Health Care
PMTCT: Prevention of Mother To Child Transmission
STD VII: Seven years of primary education in Tanzania Education System
TNMC: Tanzania Nurses and Midwives Council
UTs: Urinary Tract Infections
WHO: World Health organization

## References

[1] Anyangwe SC, Mtonga C (2007). Inequities in the global health workforce: the greatest impediment to health in sub-Saharan Africa. Int J Environ Res Public Health.

[2] Dieleman M, Toonen J, Toure H, Martineau T (2006). The match between motivation and performance management of health sector workers in Mali. Hum Resour Health.

[3] Willis-Shattuck M, Bidwell P, Thomas S, Wyness L, Blaauw D, Ditlopo P (2008). Motivation and retention of health workers in developing countries: a systematic review. BMC Health Serv Res.

[4] Montour A, Baumann A, Blythe J, Hunsberger M (2009). The changing nature of nursing work in rural and small community hospitals. Rural Remote Health.

[5] Ministry of Health CD, Gender, Elderly and Children. Human resource for health per 10,000 population by cadre as at 2015. Dar es Salaam: Ministry of Health Community Development, Gender, Elderly and Children (MoHCDGEC); 2015.

[6] Nyamtema AS, Urassa DP, Massawe S, Massawe A, Lindmark G, Van Roosmalen J (2008). Staffing needs for quality perinatal care in Tanzania. Afr J Reprod Health.

[7] TNMC. Tanzania nursing and midwifery council Data Base. Dar es Salaam: Ministry of Health Community Development, Gender, Elderly and Children (MoHCDGEC); 2013.

[8] MoHSW (2008). Basic nursing procedures. A manual for nursing practice in Tanzania.

[9] TNMC. Standards of proficiency for midwifery practice in Tanzania. Dar es Salaam: Ministry of Health Community Development, Gender, Elderly and Children (MoHCDGEC); 2007.

[10] MoHSW. Curriculum for basic Technicial certificate in nursing and midwifery NTA level 4. Dar es Salaam: Ministry of Health Community Development, Gender, Elderly and Children (MoHCDGEC); 2015.

[11] Kvale S (2009). Interviews: Learning the Craaft of Qualitative Research Interviewing. Second edition edition.

[12] WHO (2009). First global conference on task shifting.

[13] Mbindyo P, Gilson L, Blaauw D, English M (2009). Contextual influences on health worker motivation in district hospitals in Kenya. Implement Sci.

[14] Buchan J (2002). Global nursing shortages. BMJ.

[15] Manongi RN, Marchant TC, Bygbjerg IC (2006). Improving motivation among primary health care workers in Tanzania: a health worker perspective. Hum Resour Health.

[16] Almalki MJ, Fitzgerald G, Clark M (2012). Quality of work life among primary health care nurses in the Jazan region, Saudi Arabia: a cross-sectional study. Hum Resour Health.

[17] Songstad NG, Rekdal OB, Massay DA, Blystad A (2011). Perceived unfairness in working conditions: the case of public health services in Tanzania. BMC Health Serv Res.

[18] Pembe AB, Carlstedt A, Urassa DP, Lindmark G, Nystrom L, Darj E (2010). Quality of antenatal care in rural Tanzania: counselling on pregnancy danger signs. BMC Pregnancy Childbirth.

